# Issue Information

**DOI:** 10.1002/ehf2.12322

**Published:** 2019-04-29

## Abstract

No abstract is available for this article.

